# A longitudinal study on the impact of perceived stress on academic engagement among deaf college students: the moderating effect of positive emotions

**DOI:** 10.3389/fpsyg.2024.1475388

**Published:** 2024-11-15

**Authors:** Tianlin Chen, Kunjin Xu, Lan Luo, Yongfei Chen

**Affiliations:** ^1^Psychological Counseling and Guidance Center, Jiangxi University of Traditional Chinese Medicine, Nanchang, China; ^2^Student Work Department, Fuzhou Polytechnic, Fuzhou, China; ^3^School of Computer Science, Jiangxi University of Traditional Chinese Medicine, Nanchang, China

**Keywords:** perceived stress, academic engagement, positive emotions, longitudinal study, deaf college students

## Abstract

**Introduction:**

This longitudinal study sought to examine the dynamic influence of perceived stress on academic engagement among deaf college students, as well as assess the moderating role of positive emotions in this relationship. Given the unique challenges faced by deaf students in educational environments, understanding these dynamics becomes crucial for developing effective support strategies.

**Methods:**

Across a seven-month period, encompassing two semesters, 223 deaf college students were evaluated three times using the Perceived Stress Questionnaire, the Academic Engagement Questionnaire, and the Positive Emotions Questionnaire.

**Results:**

The results indicated that these students experienced high and variable levels of perceived stress, along with inadequate and similarly fluctuating levels of academic engagement. Their positive emotions were moderately low but relatively consistent. Perceived stress was found to be a negative predictor of academic engagement, while positive emotions positively predicted academic engagement. Furthermore, positive emotions acted as a moderating factor, alleviating the negative effects of perceived stress on academic engagement and serving as a protective mechanism for the academic engagement of deaf college students.

**Discussion:**

This research sheds light on the impact of perceived stress on academic engagement in deaf college students, and reveals the underlying mechanisms, contributing precious empirical evidence to further our comprehension and enhance special education services.

## 1 Introduction

The advancement of inclusive higher education has notably secured the educational rights of students with disabilities, facilitating greater access to university education for an increasingly diverse population, including students with hearing impairments (Zong and Ma, 2023). Deaf college students, characterized by auditory limitations impacting their hearing capacity (Xu et al., 2022), constitute a distinctive community within academia. These students bring unique perspectives and experiences, which sometimes manifest as alternative modes of cognitive processing, communication, and emotional intelligence due to their auditory distinctions (Catalano et al., 2021). As a result, navigating both academic environments and everyday life can present them with distinct challenges, potentially elevating their perceptions of stress (Liu, 2022).

Perceived stress refers to the psychological feelings and emotional experiences individuals undergo when they subjectively perceive and evaluate external stimuli, with tension, anxiety, and fear being the most common emotions experienced. The quantification of these emotional experiences represents the level of perceived stress (Folkman et al., 1986). Unlike the objective reality of “stress,” perceived stress is more subjective (Shalani et al., 2018). Therefore, stress and perceived stress are distinct concepts, and research on one cannot substitute for research on the other (Schaufeli et al., 2002). According to the Stress and Coping Theory (Sharma and Gupta, 2023), individuals who perceive excessive stress are inclined to adopt emotion-focused coping strategies, such as avoidance or denial of the problem, rather than problem-focused coping. While these strategies may provide temporary emotional relief, they can potentially exacerbate stress and its negative impacts in the long term. Existing research has demonstrated that high levels of perceived stress not only adversely affect individuals’ physical and mental health (Jin et al., 2022) but also severely impair their academic engagement (Rubino et al., 2009; Xie and Fan, 2014).

Academic engagement represents a positive mental attitude deeply rooted in personal learning processes, featuring enduring enthusiasm and a strong commitment to one’s studies, wherein individuals persist through obstacles and adversities (Appleton et al., 2006). According to the Self-Determination Theory (Hasniza et al., 2023), academic engagement is substantially shaped by satisfying three core psychological requirements: autonomy, proficiency, and connectedness. Fulfillment of these needs amplifies the propensity for constructive academic involvement. Yet, college students who are deaf may confront specific hurdles stemming from auditory limitations, which have the potential to amplify feelings of stress and diminish academic engagement (Deci and Ryan, 2000); these circumstances underscore the importance of recognizing and addressing unique support needs within this student demographic (Deci and Ryan, 2000). Studies by Yang (2015) and Cao and Liu (2017) found a significant negative correlation between perceived stress and academic engagement; higher perceived stress leads to a greater risk of learning burnout, thereby decreasing academic engagement. These studies provide empirical support for the negative relationship between perceived stress and academic engagement. However, existing research (Liu, 2020; Xie and Fan, 2014) primarily focuses on general college students and rarely involves those with hearing impairments. Furthermore, the exact mechanisms through which perceived stress affects academic engagement remain unresolved.

Meng (1988) suggests that positive emotions are innate subjective experiences derived from satisfying needs, enhancing an individual’s drive and aiding in overcoming difficulties, achieving self-improvement, and maintaining well-being. These emotions make actions more proactive, persistent, and effective. There is a strong connection between positive emotions and academic engagement: emotions like happiness, interest, and satisfaction are linked to greater academic engagement and can positively predict students’ learning engagement (Yan, 2016; King and Gaerlan, 2014; Appleton and Christenson, 2008). Additionally, research (Guo and Wang, 2007) indicates that positive emotions can moderate the impact of stress on outcomes. According to the Conservation of Resources Theory, facing stress can deplete psychological resources, leading to negative emotions and coping behaviors that negatively affect academic engagement if not replenished (Hobfoll, 2001). However, effective replenishment of these resources can alter this situation. Fredrickson’s (2003) Broaden-and-Build Theory posits that positive emotions catalyze beneficial thoughts and actions, nurturing confidence, hope, and optimism, which construct adaptive resources for handling change and stress. Based on this, we hypothesize that positive emotions moderate the effect of perceived stress on academic engagement.

In summary, existing research predominantly explores the relationship between perceived stress and academic engagement, as well as that between positive emotions and academic engagement, through cross-sectional studies, offering substantial insights. However, since these studies collect data at a single point in time, they can only reveal correlations between variables without clarifying causal relationships—a limitation acknowledged in the literature. Moreover, studies (Cao and Ji, 2024; Liu et al., 2024) highlight that an individual’s psychological state or traits may fluctuate during certain critical stages. Given this insight, employing a longitudinal design appears more suitable for examining individuals in developmental phases. Therefore, this study adopts a three-wave longitudinal design spanning one academic year across two semesters, complemented by cross-lagged panel analyses, leading us to propose the following hypotheses: Perceived stress significantly negatively predicts academic engagement in deaf college students. Positive emotions play a significant moderating role longitudinally in the relationship between perceived stress and academic engagement among deaf college students. This research endeavor aims to elucidate the impact of perceived stress on academic engagement and uncover the underlying mechanisms. By doing so, it will contribute empirical evidence to enhance academic engagement in deaf college students and refine special education services.

## 2 Methods

### 2.1 Participants

Using stratified random sampling, we selected deaf college students ranging from first-year to fourth-year from multiple institutions in Fuzhou, Nanjing, Tianjin, and other locations. The inclusion criteria were: certified hearing disability, current enrollment in school, informed consent, and voluntary participation. Students were excluded if they were out of school due to suspension or other reasons, or if they had both a hearing disability and another disability. Data collection was conducted three times: the first (T1) in late September 2023, the second (T2) in mid-January 2024, and the third (T3) in early May 2024, with a seven-month interval between each, spanning two semesters within an academic year.

According to Wu (2010), the recommended sample size for factor analysis should be 5 to 10 times the number of questionnaire items. Given that this study comprised 58 items, the sample size was set at 200 to 400 participants. Specifically, T1 surveyed 293 participants, T2 surveyed 289, and T3 surveyed 271. After the survey, based on T3, we excluded 27 questionnaires due to incomplete tests (participants did not complete all three tests through student IDs matching) and 21 questionnaires due to excessively short completion times (less than 60 s, which is three standard deviations below the average of 90.03 ± 10.01 s for the three tests). This resulted in a final sample of 223 valid participants. The valid participants included 124 males (55.61%) and 99 females (44.39%); 186 from urban areas (83.41%) and 37 from rural areas (16.59%); 196 only children (87.89%) and 27 non-only children (12.11%); 77 freshmen (34.53%), 64 sophomores (28.70%), and 82 juniors and seniors combined (36.77% due to fewer numbers); 191 with Level I hearing disability (85.65%) and 32 with Level II and above combined (14.35% due to fewer numbers in Levels II, III, and IV).

### 2.2 Survey tools

#### 2.2.1 General information questionnaire

This questionnaire included information about the participant’s student IDs, gender (male, female), residence (urban or rural), only child (yes or no), grade (freshman, sophomore, junior, senior), and degree of hearing disability (Level I, II, III, IV). Among these, student IDs were used to match the three tests, while demographic factors such as gender, birthplace, status as an only child, grade, and degree of hearing impairment may influence differences in perceived stress, academic engagement, and positive emotions among the study participants (e.g., Cao and Liu, 2017; Jin et al., 2022; Liu, 2022; Xie and Fan, 2014). Consequently, these demographic variables were also treated as control variables in the subsequent research.

#### 2.2.2 Perceived stress questionnaire

Using the Perceived Stress Questionnaire developed by Yang and Huang (2003). The questionnaire consists of 14 items, with items 4, 5, 6, 7, 9, 10, and 13 being reverse scored. It employs a Likert-5 point scoring method, with higher scores indicating higher levels of perceived stress. The scale is widely used and has good reliability and validity. We tested the reliability of the scale, and the Cronbach’s α coefficients for T1, T2, and T3 were 0.851, 0.826, and 0.873, respectively.

#### 2.2.3 Academic engagement questionnaire

Using the Academic Engagement Questionnaire developed by Fang et al. (2008). The questionnaire contains 17 items, using a Likert-7 point scoring method, with higher scores indicating higher levels of academic engagement. The scale has been adopted by many scholars and has good reliability and validity. We tested the reliability of the scale, and the Cronbach’s α coefficients for T1, T2, and T3 were 0.844, 0.827, and 0.855, respectively.

#### 2.2.4 Positive emotions questionnaire

Using the Positive Emotions Questionnaire developed by Qiu et al. (2008). The questionnaire includes 9 descriptive items related to positive emotions, employing a Likert-5 point scoring method, with higher scores representing higher levels of positive emotions. The questionnaire is widely used and has good reliability and validity. We tested the reliability of the scale, and the Cronbach’s α coefficients for T1, T2, and T3 were 0.911, 0.893, and 0.938, respectively.

### 2.3 Procedure

The research was conducted through the Questionnaire Star platform,^1^ a widely utilized tool in China for data collection, questionnaire administration, and various research endeavors. This platform provides a user-friendly interface alongside comprehensive features for designing, disseminating, and analyzing questionnaires. The designed questionnaires were imported into the Questionnaire Star platform and converted into QR codes. Teachers responsible for working with deaf college students at relevant schools were entrusted to display the survey on multimedia screens during class meetings, allowing the students to complete the questionnaire with informed consent. Prior to the survey, teachers conducting the survey were trained online to understand the entire measurement process. The survey was conducted anonymously (but required student ID) to ensure the authenticity of the data collected. Each question was mandatory, and each IP address could submit only one questionnaire. The procedures for T1, T2, and T3 were consistent. After the survey, the questionnaires collected from the three tests were sorted, and invalid questionnaires that could not be matched (by student *ID*, requiring completion of all three tests) or had excessively short completion times (three standard deviations below the average time for the three tests) were eliminated, resulting in valid questionnaires from the three tests.

### 2.4 Data processing

The valid data of 223 × 3 sets were uniformly coded and analyzed using *SPSS 25.0* and *AMOS 21.0*. main analyses included normality testing, common method bias testing, repeated measures *ANOVA*, spearman correlation analysis, cross-lagged model testing, and moderation effect testing.

## 3 Results

### 3.1 Normality test and common method Bias test

The K-S test (Kolmogorov–Smirnov test) method was used to examine the normality of the distribution for the data collected from the three scales. Among the 9 sets of data from T1, T2, and T3 tests, the absolute values of kurtosis ranged from 0.461 to 1.284, and the absolute values of skewness ranged from 0.061 to 0.679. Since the requirements for normality testing are strict and usually difficult to meet, if the absolute value of kurtosis is less than 10 and the absolute value of skewness is less than 3, then although the data are not absolutely normal, they can be considered as approximately normally distributed (Drezner et al., 2010). Therefore, the data in this study can be considered to be approximately normally distributed.

The Harman single-factor method was used to test for common method bias in the data collected from the three scales. During the T1, T2, and T3 cycles, there were 6, 5, and 5 factors with eigenvalues greater than 1, respectively, and the variance explained by the first factor was 23.67, 26.53, and 25.932%, respectively (all below the critical value of 40%; Podsakoff et al., 2003). These results indicate that the questionnaire data does not converge into a single factor, and different variables within the data are not influenced by a systematic bias. Therefore, we conclude that there is no significant common method bias in our data.

### 3.2 Dynamic analysis of the key variables

The results of this study show that the scores for perceived stress (*M* ± *SD*) were 0.910 ± 15.685, 54.848 ± 13.389, 51.901 ± 14.271 at T1, T2, and T3, respectively, all significantly higher than the theoretical median of 42.00, indicating that deaf college students generally experienced severe perceived stress. The scores for academic engagement were 56.794 ± 25.237, 51.928 ± 28.623, and 53.861 ± 24.920 at T1, T2, and T3, respectively, all significantly lower than the theoretical median of 68.00, suggesting that overall, there was a lack of academic engagement among deaf college students. The scores for positive emotions were 23.395 ± 10.878, 22.300 ± 10.580, and 224.175 ± 10.498 at T1, T2, and T3, respectively, all lower than the theoretical median of 27.00, indicating that the overall level of positive emotions among deaf college students was moderately low.

Repeated measures *ANOVA* (Tukey’s *HSD*) was performed on the data from T1, T2, and T3 measurements (Table 1): The scores for stress perception exhibit dynamic fluctuations within the T1 to T3 time points. Specifically T2 > T3 > T1 (*F* = 7.447, *p* < 0.001). The scores for academic engagement also demonstrate dynamic fluctuations across the T1 to T3 time points, with the specific pattern being T1 > T3 > T2 (*F* = 3.848, *p* < 0.05). However, the fluctuations in positive emotions from T1 to T3 did not reach a significant level (*F* = 2.124, *p* = 0.121>0.05), suggesting that they can be considered relatively stable.

**Table 1:** Repeated measures ANOVA results.

Variable	Effect	Square sum	*df*	Mean square	*F*	*p*	Partial η^2^	*HSD*
Perceived stress	Intercept	1847660.634	1	1847660.634	4891.020^***^	0.000	0.957	-
	Time (T)	1870.514	2	935.257	7.447^***^	0.001	0.032	T2>T3>T1
Academic engagement	Intercept	1964869.262	1	1964869.262	1501.039^***^	0.000	0.871	-
	Time (T)	2676.685	2	1338.342	3.484^*^	0.032	0.015	T1>T3>T2
Positive emotions	Intercept	355051.934	1	355051.934	1472.692^***^	0.000	0.869	-
	Time (T)	181.707	2	90.854	2.124	0.121	0.009	-

^*^p < 0.05, ^*^p < 0.01, ^**^p < 0.001, the same applies below.

### 3.3 Correlation analysis of the key variables

The results of spearman correlation analysis indicate (Table 2): At T1, T2, and T3, perceived stress is significantly negatively correlated with academic engagement and positive emotions (all *p* < 0.01), while positive emotions is significantly positively correlated with academic engagement (*p* < 0.01). Additionally, there are significant positive correlations between perceived stress scores, academic engagement levels, and positive emotions at all three time points (all *p* < 0.01).

**Table 2:** Correlation analysis of perceived stress, academic engagement, and positive emotions (*n* = 223).

	1	2	3	4	5	6	7	8	9
1. Perceived Stress (T1)	1								
2. Perceived Stress (T2)	0.365^**^	1							
3. Perceived Stress (T3)	0.459^**^	0.338^**^	1						
4. Positive Emotions (T1)	−0.282^**^	−0.215^**^	−0.300^**^	1					
5. Positive Emotions (T2)	−0.500^**^	−0.367^**^	−0.318^**^	0.592^**^	1				
6. Positive Emotions (T3)	−0.445^**^	−0.278^**^	−0.565^**^	0.528^**^	0.535^**^	1			
7. Academic Engagement (T1)	−0.546^**^	−0.398^**^	−0.565^**^	0.300^**^	0.380^**^	0.488^**^	1		
8. Academic Engagement (T2)	−0.362^**^	−0.243^**^	−0.406^**^	0.391^**^	0.296^**^	0.508^**^	0.417^**^	1	
9. Academic Engagement (T3)	−0.454^**^	−0.471^**^	−0.366^**^	0.264^**^	0.386^**^	0.383^**^	0.516^**^	0.388^**^	1

### 3.4 Cross-lagged analysis of the key variables

To examine the dynamic causal relationships among perceived stress, positive emotion, and academic engagement of deaf college students, we conducted cross-lagged regression analyses on the basis of preliminary correlational analyses. Two cross-lagged models were constructed: one depicting the interaction between perceived stress and academic engagement, and another illustrating the interaction between positive emotion and academic engagement.

**Figure 1:**
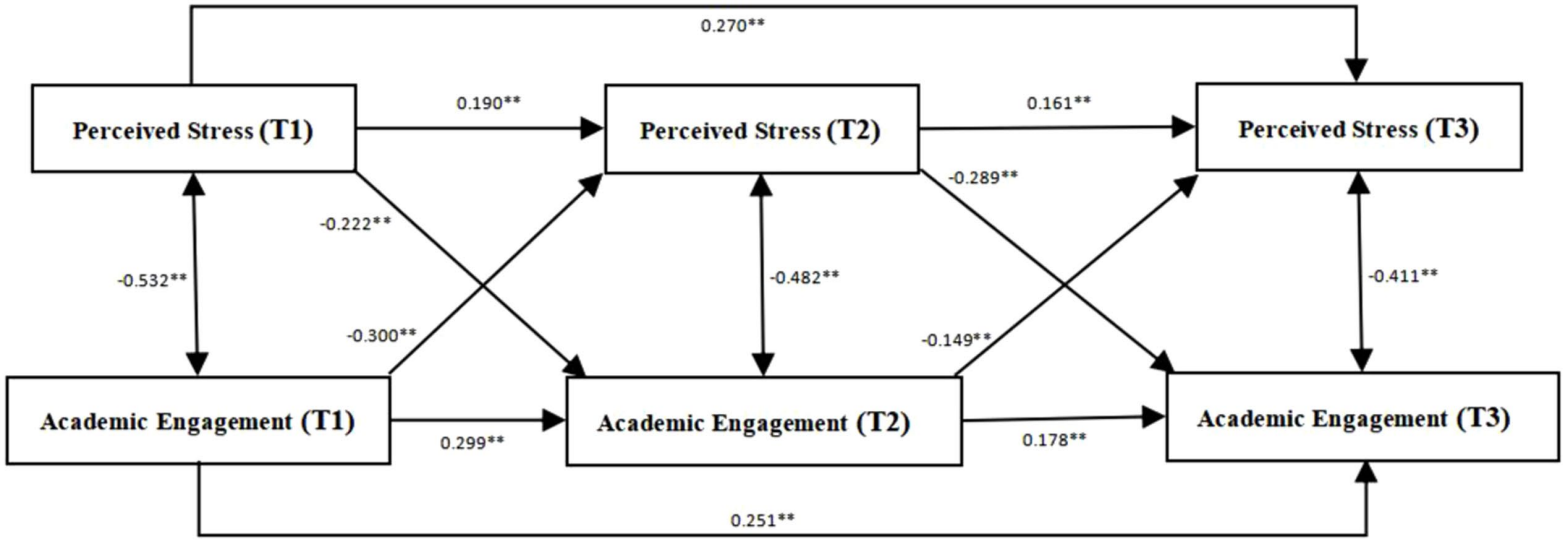
Cross-lagged model of perceived stress and academic engagement.

The bias-corrected bootstrap test, after resampling 1,000 times (as shown in Figure 1), reveals that perceived stress (T1) significantly predicts perceived stress (T2) and perceived stress (T3; with *β* values of 0.190 and 0.270, respectively, both with *p* < 0.01). Additionally, perceived stress (T2) significantly positively predicts perceived stress (T3; *β* = 0.161, *p* < 0.01). Conversely, perceived stress (T1) significantly negatively predicts academic engagement (T2; *β* = −0.222, *p* < 0.01), and perceived stress (T2) significantly negatively predicts academic engagement (T3; *β* = −0.289, p < 0.01). Simultaneously, academic engagement (T1) significantly negatively predicts perceived stress (T2; *β* = −0.300, *p* < 0.01), and academic engagement (T2) significantly negatively predicts perceived stress (T3; *β* = −0.149, *p* < 0.01). This indicates a reciprocal predictive causal relationship between perceived stress and academic engagement.

**Figure 2:**
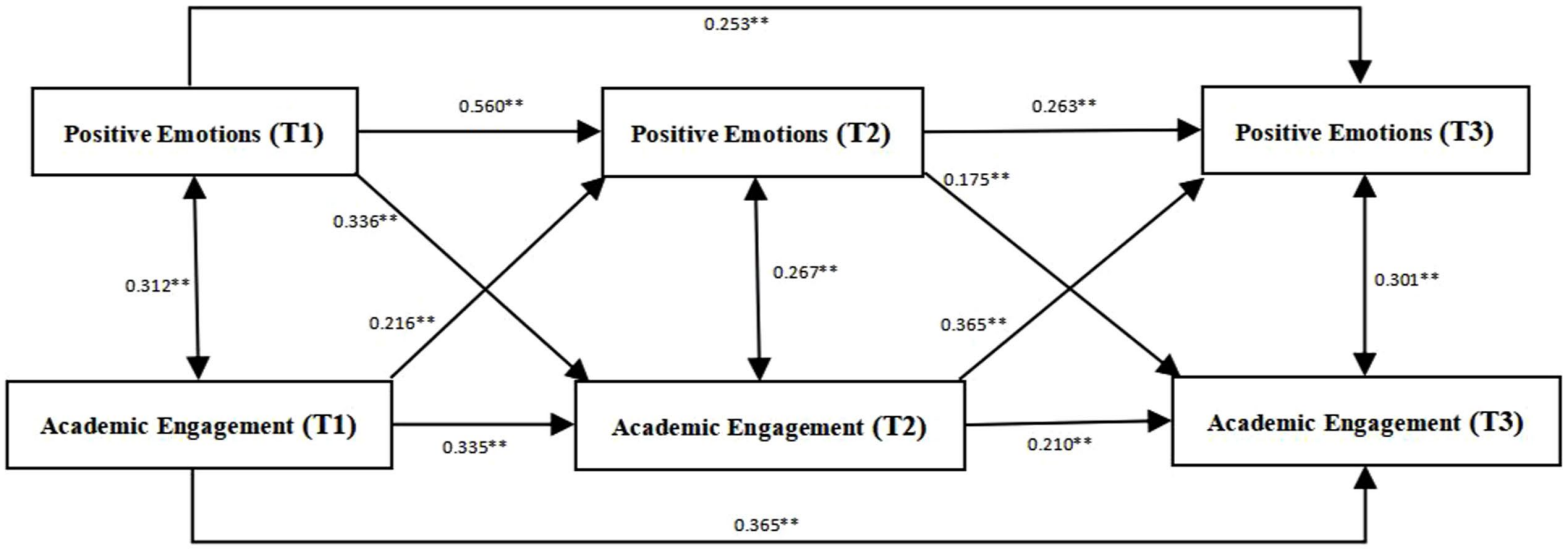
Cross-lagged model of positive emotions and academic engagement.

Figure 2 shows that positive emotions (T1) significantly positively predict positive emotions positive emotions (T2) and positive emotions (T3; with β values of 0.560 and 0.253, respectively, both with p < 0.01). Furthermore, positive emotions (T2) significantly positively predict positive emotions (T3; *β* = 0.263, *p* < 0.01). Positive emotions (T1) also significantly positively predict academic engagement (T2; *β* = 0.336, *p* < 0.01), and positive emotions (T2) significantly positively predict academic engagement (T3; *β* = 0.175, *p* < 0.01). Concurrently, academic engagement (T1) significantly positively predicts positive emotions (T2; *β* = 0.216, *p* < 0.01), and academic engagement (T2) significantly positively predicts positive emotions (T3; *β* = 0.365, *p* < 0.01). This suggests a reciprocal predictive causal relationship between positive emotions and academic engagement.

### 3.5 Longitudinal moderating effect analysis

To examine the longitudinal moderating effect of positive emotions among deaf college students on the relationship between perceived stress and academic engagement, we used academic engagement (T3) as the dependent variable, perceived stress (T1) as the predictor variable, and positive emotions (T2) as the moderator variable (both were centered). An interaction term “perceived stress (T1) × positive emotions (T2)” was formed, and hierarchical regression analysis was conducted. In step 1, demographic variables (gender, place of origin, only-child status, grade level, disability rating) were included as control variables in the equation. In step 2, both perceived stress and positive emotions were added to the equation. In step 3, the “perceived stress (T1) × positive emotions (T2)” interaction term was incorporated into the equation.

The results showed (Table 3): perceived stress (T1) negatively predicted academic engagement (T3; *β* = −0.398, *p* < 0.001), and positive emotions (T2) positively predicted academic engagement (T3; *β* = 0.227, *p* < 0.001). The moderating effect of positive emotions (T2) on the relationship between perceived stress (T1) and academic engagement (T3) among deaf college students was significant (*β* = 0.237, *p* < 0.001), explaining 5.1% of the variance in academic engagement (T3; △*R^2^* = 0.051). Wen and Ye (2014) suggested that a variance change of more than 2% has substantive significance. Therefore, the moderating effect of positive emotions (T2) on the relationship between perceived stress (T1) and academic engagement (T3) among deaf college students has substantive meaning.

**Table 3:** Longitudinal moderating effect analysis of positive emotions (T2) on the relationship between perceived stress (T1) and academic engagement (T3; *n* = 223).

	Model					Model					Model				
	*B*	*SE*	*t*	*p*	*β*	*B*	*SE*	*t*	*p*	*β*	*B*	*SE*	*t*	*p*	*β*
Constant	39.321	9.331	4.214^***^	0.000	-	40.704	9.189	4.430^***^	0.000	-	39.170	8.894	4.404^***^	0.000	-
Genders	4.593	3.165	1.451	0.148	0.092	4.969	3.115	1.595	0.112	0.099	5.306	3.013	1.761	0.080	0.106
Residence	5.150	4.343	1.186	0.237	0.077	4.031	4.289	0.940	0.348	0.060	5.696	4.169	1.366	0.173	0.085
Only child	−4.368	4.781	−0.914	0.362	−0.057	−4.345	4.702	−0.924	0.356	−0.057	−4.228	4.547	−0.930	0.354	−0.055
Grade	3.213	1.771	1.814	0.071	0.109	3.077	1.742	1.766	0.079	0.104	4.018	1.701	2.362^*^	0.019	0.136
Degree of hearing disability	0.246	2.552	0.097	0.923	0.006	−0.055	2.512	−0.022	0.982	−0.001	−0.510	2.432	−0.210	0.834	−0.012
Perceived Stress (T1)	−11.311	1.501	−7.535^***^	0.000	−0.454	−9.047	1.672	−5.411^***^	0.000	−0.363	−9.907	1.631	−6.074^***^	0.000	−0.398
Positive Emotions (T2)						4.791	1.660	2.886^**^	0.004	0.192	5.647	1.620	3.487^***^	0.001	0.227
perceived stress (T1) × positive emotions (T2)											5.112	1.282	3.988^***^	0.000	0.237
*R* ^2^	0.233					0.261					0.312				
△*R* ^2^	0.233					0.029					0.051				
*F* 值	*F* (6,216) = 10.918^***^					*F* (1,215) = 8.329^**^					*F* (1,214) = 15.901^***^				

Dependent variable: academic engagement (T3).

**Figure 3:**
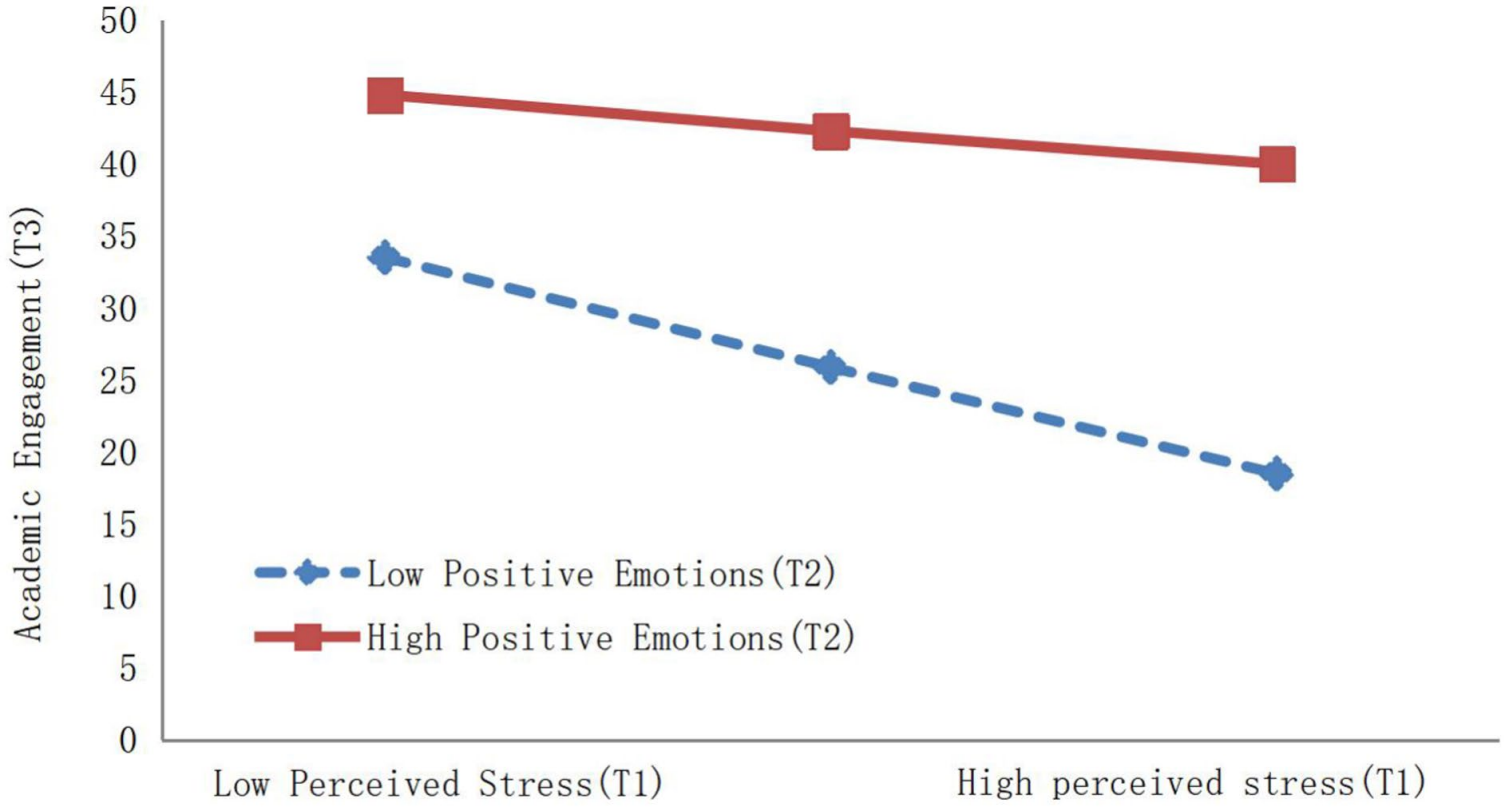
The longitudinal moderating effect of positive emotions (T2) on the relationship between perceived stress (T1) and academic engagement (T3).

To further analyze the impact of perceived stress (T1) on academic engagement (T3) among deaf college students with different levels of positive emotions, the scores of positive emotions (T2) were divided into high positive emotions group and low positive emotions group based on M ± SD, followed by simple slope tests. The results showed (Figure 3): Compared to the low positive emotions group (*β* = −25.654, *t* = −20.283, *p* < 0.001), the negative influence of perceived stress (T1) on academic engagement (T3) was smaller in the high positive emotions group (*β* = −10.148, *t* = −10.291, *p* < 0.001). This indicates that as the level of positive emotions increases, the negative impact of perceived stress on academic engagement among deaf college students decreases, and the higher the level of positive emotions, the stronger this moderating effect becomes.

## 4 Discussion

### 4.1 Deaf college students experience a significantly higher level of perceived stress, which exhibits dynamic fluctuations

This study found that deaf college students have a severe sense of perceived stress, which contrasts with the research of Yuan and Fan (2021) showing that deaf college students’ perceived stress is at a moderate level. The reason for this discrepancy may be due to different sampling methods. Previous studies have shown that perceived stress among ordinary college students is at a high level (Liu, 2020; Xie and Fan, 2014). Indeed, deaf college students navigate a range of experiences similar to those encountered by other students in areas like academics, social interactions, emotional well-being, and career planning (Li, 2022). However, given their unique position, they may also grapple with internalized stigma, fluctuating self-esteem, feelings of being on the periphery, perceptions of bias, and proactive social engagement challenges, which require tailored support systems and interventions (Li, 2022). The Stress and Coping Theory (Sharma and Gupta, 2023) suggests that an individual’s cognitive evaluation of a stressor is a crucial factor influencing their perceived stress. Due to their physiological deficiencies, deaf college students may be more prone to negatively evaluate and cope with a series of issues that exceed those faced by ordinary college students, thereby accumulating negative emotions and resulting in a high perceived stress.

Deaf college students’ perceived stress, although severe, also shows significant periodic fluctuations: the highest perceived stress occurs around mid-January, followed by early May, and the lowest in late September. This may be related to China’s semester system and examination arrangements. January is usually the final exam period for the fall semester, during which deaf college students need extra time and energy to prepare for exams, especially if they rely on sign language interpretation or other assistive technologies, which may increase their perceived stress. Although early May is the middle of the spring semester, it is also the time to prepare for final exams, possibly leading to some perceived stress among deaf college students. In contrast, late September is just the beginning of a new semester with relatively light academic loads, thus causing less perceived stress among deaf college students.

### 4.2 Deaf college students have inadequate academic engagement, which also exhibits dynamic fluctuations

Deaf college students often exhibit varying degrees of academic engagement, which aligns with findings reported by Liu (2019). It is crucial to understand that reduced academic engagement is not indicative of a lack of motivation or ability but rather a complex interplay between personal, educational, and environmental factors. Deaf college students frequently contend with unique challenges, including language accessibility issues, hearing-related communication barriers, and a lack of tailored instructional methods designed to meet diverse learning needs (Guo, 2018; Yuan and Fan, 2021). Furthermore, the absence of fully equipped learning support infrastructures and accessible learning environments significantly impedes engagement levels. These students’ experiences reflect broader systemic issues that require targeted interventions to promote inclusive education practices.

Deaf college students’ academic engagement fluctuates significantly at different times: the highest level of academic engagement is observed in late September, followed by early May, and the lowest in mid-January. This phenomenon may be related to factors such as semester cycles, individual motivation levels, and psychological states. Late September marks the beginning of a new semester when deaf college students typically encounter new course content and projects. They may be filled with anticipation for new courses and learning objectives, with more energy and a positive mindset to face academic challenges, thus exhibiting high academic engagement. As the semester progresses, this initial enthusiasm may gradually wane. By the end of the semester (mid-January), prolonged academic pressure, long hours of study, and exam preparation may cause deaf college students to feel fatigued and burnt out, leading to a decrease in academic engagement. However, during the middle of the semester (early May), as the study time for deaf college students is not long, their learning enthusiasm may still be present, and academic burnout is relatively not so serious, hence maintaining a certain level of academic engagement.

### 4.3 Deaf college students report moderately low levels of positive emotions, which maintain relative stability

Deaf college students exhibit moderate to below average positive emotions, which may stem from unique challenges they face in areas such as social support, interpersonal communication, coping resources, and societal cultural attitudes. First, insufficient social support (Yuan and Fan, 2021). Deaf college students may face communication barriers in their daily lives, limiting their ability to establish deep social connections with others. A lack of adequate social support networks can lead to decreased positive emotions. According to the Social Support Theory (Jia and Cheng, 2024), good social support can promote individual positive emotions, while the lack of a sufficient social support network may lead to a decrease in positive emotions. Second, communication challenges (Xu et al., 2022). Deaf college students may encounter difficulties in daily communication, which can lead to misunderstandings, frustration, and feelings of isolation. These negative experiences may weaken their positive emotions. Communication barriers may also make them feel frustrated when seeking help or expressing needs, further affecting their emotional state. Third, limited coping resources (Liu, 2022). Deaf college students may lack effective coping strategies or resources when dealing with academic and life challenges. The resource conservation theory states that when individuals perceive a loss or threat to their resources, they experience stress and negative emotions. Deaf college students may lack sufficient time, energy, or expertise to effectively cope with challenges, resulting in low positive emotions. Fourthly, the influence of societal and cultural norms (Yuan and Fan, 2021) plays a pivotal role in shaping the emotional health of deaf students. Prevailing societal beliefs and cultural values concerning disability can significantly impact the way deaf individuals perceive themselves and their place within society. When society at large embraces stigmatizing viewpoints or fails to uphold principles of equality for persons with disabilities, this can understandably lead deaf students to sense a diminishment of their own value and experience feelings of isolation. Such dynamics may pose obstacles to nurturing a robust reservoir of positive emotions.

The observed consistency in positive emotions among deaf college students over the course of the study could stem from a few pivotal elements. Initially, considering the study timeframe encompassed merely 8 months, a rather condensed period, shifts in the students’ positive emotions would probably be too subtle to detect. Additionally, the demographic studied comprised young adults who usually have cemented their social identities and delineated trajectories for their social growth. This stage of life is marked by reduced mood swings, as per observations by Kwon et al. (2018). Moreover, positive emotions are chiefly molded by personal traits, lived experiences, mental health standing, and surrounding environments. Typically, these influencers experience scant transformation in everyday life scenarios, hence leading to the observed equilibrium in an individual’s positive affective state, as underscored by Yan (2016).

### 4.4 Deaf college students’ perceived stress negatively predicts academic engagement, while positive emotions positively predicts academic engagement

Current studies do not explore perceived stress and academic engagement link in deaf college students. Yet, findings indicate: Perceived stress negatively impacts academic engagement among deaf students. This study revealed that perceived stress among deaf college students negatively predicts academic engagement, which is consistent with the research of Hua et al. (2016) on ordinary college students. This means that the greater the perceived stress felt by deaf college students, the less they engage in academics. According to Self-Determination Theory (Hasniza et al., 2023), when an individual’s three basic psychological needs of autonomy, competence, and relatedness are met, they are more likely to exhibit positive academic engagement. For deaf college students, perceived stress may undermine their autonomy in learning, diminish their sense of competence, and reduce their sense of belonging, thereby affecting their learning motivation. This, in turn, leads to their inability to fully devote themselves to their studies, ultimately resulting in a decrease in academic engagement.

Positive emotions positively predicts academic engagement, meaning that the higher an individual’s positive emotions, the higher their level of academic engagement. This finding is consistent with the research of Ma et al. (2019). Previous studies have pointed out that in academia, positive emotions enhances students’ long-term motivation to pursue learning goals and facilitates active engagement in learning (Claro and Dweck, 2016). And this study posits that positive emotions, acting as internal assets, enhance learning engagement by cultivating interest, focus, resilience, and optimism. They boost confidence in academic pursuits, driving higher educational involvement. Isen (2002) found that compared to neutral states, individuals with positive emotions exhibit higher creativity and efficiency in problem-solving, which is conducive to academic engagement. Liu et al. (2024) posit that, compared to students who are less academically engaged, students in this state are more likely to adopt mastery-oriented goals for their academics, thereby tending to score higher and exhibit overall superior academic performance. Therefore, this study, along with previous research, demonstrates that positive emotions can positively predict the academic engagement levels of deaf college students.

### 4.5 Positive emotions plays a significant moderating role in the relationship between deaf college students’ perceived stress and academic engagement

This study showed that positive emotions plays a significant moderating role in the relationship between deaf college students’ perceived stress and academic engagement. The research outcome unveils the inner workings behind how perceived stress affects academic engagement in deaf college students. It points out that positive emotions play a crucial role as a mediator, operating like a cushion or regulator. This occurs between handling stress and engaging in academic endeavors among deaf college students, highlighting its significance in buffering or moderating these processes. Drawing from Fredrickson’s (2003) Broaden-and-Build Theory of Positive Emotions, this investigation proposes that the regulatory influence of positive emotions hinges largely on their capacity to broaden perspectives, build resources, and alleviate distress. Individuals endowed with robust positive emotions tend to widen their cognitive and action horizons, construct personal reservoirs of mental strength, and temper, rectify, and heal negative sentiments. As a result, they demonstrate enhanced focus, cognitive agility, and flexibility when addressing problems, equipped with substantial coping mechanisms to counteract the detrimental feelings spawned by pressure. This equips them to concentrate more intently and engage fully in their studies. In contrast, those with deficient positive emotions find themselves bereft of adequate coping strategies, manifesting passive and rigid approaches to problem-solving, and being more susceptible to negative emotions. Such predispositions trigger adverse reactions and a propensity to evade stress, culminating in a dearth of motivation for learning. Ultimately, this leads to a pronounced negative fallout from perceived stress on academic engagement. Thus, positive emotions can help deaf college students more effectively cope with academic stress and maintain positive engagement in their studies.

## 5 Summary

### 5.1 Conclusion

This study employed empirical methods to explore the relationship between deaf college students’ perceived stress and their academic engagement, revealing that positive emotions played a moderating role in this relationship, a finding of great importance for research in the field of special education. The study yields the following conclusions:

Perceived stress among deaf college students was severe and exhibited dynamic fluctuations, while academic engagement was insufficient and also showed dynamic patterns, and positive emotions were found to be moderately low but remained relatively stable.

Perceived stress was identified as a negative predictor of academic engagement, whereas positive emotions positively predicted academic engagement.

Positive emotions plays a significant moderating role in the relationship between perceived stress and academic engagement among deaf college students. They help mitigate the negative effects of perceived stress on academic engagement, thereby offering a protective function for the academic engagement of deaf college students.

### 5.2 Research limitations

Although this study is rich in content and offers valuable insights for reference in special education, it nonetheless has certain limitations that may impact the accuracy and generalizability of its findings.

Firstly, as the research subject was a special group of deaf college students, obtaining a sufficiently large sample size posed certain challenges. The sample size of this study is relatively small, consisting of merely 223 participants, and its source diversity is insufficient. These factors may lead to limitations in the representativeness and statistical power of the research findings. The insufficiency of the sample size might prevent the study from fully detecting certain effects or introduce considerable uncertainties when inferring overall conditions. Secondly, the homogeneity of the study sample was high, with most participants (85.65%) belonging to the mildly hearing-impaired category of Grade I disability, and the sample mainly coming from specific schools and regions. This centralization of sample sources might lead to the research results reflecting the characteristics of specific groups or regions rather than the general situation of the entire deaf college student population. Consequently, the extrapolative and generalized capabilities of the research results might be constrained. Thirdly, the study has not sufficiently taken into account the impact of cultural differences and varied educational environments on students. To some extent, this oversight will result in biased conclusions and restrict the cross-cultural applicability and value of the research findings.

To address these limitations, future research could adopt the following measures to overcome them: (1) Expand the sample size: Collaborate across multiple centers or conduct cross-regional surveys to collect more data, thereby enhancing the research’s statistical power and the reliability of the results. Additionally, modern technological means such as online surveys could be utilized to broaden data collection channels and attract more eligible participants to join the study. (2) Increase sample diversity: In future research designs, efforts should be made to include deaf college students with different disability levels, from different regions, and with varying educational backgrounds, ensuring that the sample represents a wider range of populations. Enhancing the heterogeneity of the sample can strengthen the general applicability of the research findings. (3) Combine qualitative and quantitative research: In addition to quantitative research, qualitative research methods such as interviews and focus groups could be combined to delve deeper into the subjective experiences and coping strategies of deaf college students facing academic stress, providing richer data and deeper insights. Through these improvement measures, future research can comprehensively and deeply explore the relationship between deaf college students’ perceived stress, positive emotions, and academic engagement, offering a more solid theoretical foundation and empirical support for special education practice. (4) In order to enhance the comprehensiveness and generalizability of the research, it is imperative to incorporate diverse perspectives. By conducting comparative analyses, we can identify the potential influence of cultural and educational system factors on the relationships between core variables. This approach will ensure that the outcomes of our research serve to improve educational practices across the globe more effectively.

### 5.3 Implications for research

Based on the negative predictive effect of perceived stress on academic engagement among deaf college students, one key strategy to enhance their academic engagement is to alleviate their level of perceived stress. Firstly, it is essential to regularly assess the perceived stress of deaf college students. Given their heightened perceived stress, it is recommended that universities conduct comprehensive screenings and assessments of their mental health status through regular psychological surveys, counseling sessions with advisors, and information gathering by mental health committee members. For those deaf college students exhibiting significant perceived stress, targeted attention should be given, and timely provision of appropriate psychological counseling, stress reduction training, or emotional support should be provided. Secondly, it is crucial to establish a robust support system for deaf college students. Universities should form specialized support teams comprising mental health experts, academic advisors, and hearing specialists to provide comprehensive support and guidance for these students; offer advanced hearing assistance devices and technologies, such as hearing aids, real-time captioning services, and audio amplifiers, to help deaf college students better participate in classroom learning and social activities, alleviating academic and life pressures; create an accessible environment: build barrier-free campus facilities, including accessible classrooms, dormitories, and public spaces, to ensure that deaf college students can easily access and utilize these areas, reducing their psychological stress; and strengthen teacher training by providing teachers with targeted training and guidance for deaf college students, helping them better understand their needs and employ more effective teaching methods and strategies to reduce the perceived stress of deaf college students in their studies. Thirdly, it is vital to emphasize cultivating the self-psychological regulation abilities of deaf college students. “Give a man a fish, and you feed him for a day; teach a man to fish, and you feed him for a lifetime.” It is recommended that in mental health education classes, deaf college students should learn basic methods and techniques to alleviate psychological confusion, regulate psychological conflicts, and relieve stress, fostering their self-psychological regulation abilities; outside the classroom, outdoor expansion training should also be conducted to cultivate their ability to withstand pressure and endure setbacks. Finally, leveraging AI technology can significantly enhance stress management for deaf college students. Utilizing AI-powered applications to transcribe speech-to-text in real-time during classroom lectures, meetings, and daily conversations facilitates better comprehension and engagement for deaf students, mitigating the pressure stemming from missed information. Moreover, employing VR technology to create immersive relaxation settings, such as tranquil natural landscapes or guided meditation sessions, can help relieve tension and anxiety among deaf students. Hence, AI does not merely alleviate the immediate pressures faced by deaf undergraduates in their personal and academic lives; it fosters a more inclusive and responsive support infrastructure. This ensures that they have equitable opportunities and maintain a healthier mindset throughout their educational journey.

Given that the level of positive emotions can moderate the negative impact of deaf college students’ perceived stress on academic engagement, promoting their academic engagement requires elevating their positive emotions as another viable strategy. This study indicates that deaf college students’ positive emotions is relatively stable. Therefore, merely relying on natural growth and development to improve their positive emotions level is challenging and necessitates external intervention. Firstly, both schools and families should actively create an environment conducive to generating positive emotions, providing timely positive feedback to deaf college students for their efforts and achievements to stimulate and enhance their positive life and learning experiences, fostering their ability to autonomously regulate and control their emotions, thereby promoting the elevation of their positive emotions levels. Secondly, it is advised that schools design personalized curricula and learning tasks, enabling deaf college students to achieve success progressively, gain successful learning experiences, and bolster their confidence and motivation to learn. Thirdly, provide deaf college students with social skills training to help them build and maintain interpersonal relationships and strengthen their social support network. Encourage and guide deaf college students to actively participate in various cultural and sports activities, which can not only overcome feelings of inferiority and boost confidence through appropriate self-expression but also improve perceived discrimination and obtain broader interpersonal support by interacting with ordinary college students, strengthening self-identity, and subsequently enhancing their positive emotions levels. Thirdly, AI has paved new roads in enhancing the positive emotional experiences of deaf college students. It achieves this through boosting self-awareness with emotion recognition and feedback systems, stimulating interest via customized games and interactive content, accumulating a sense of accomplishment with personalized achievement rewards, diminishing loneliness and expanding social networks with virtual companions and optimized social platforms, fostering inner peace and happiness through AI-guided mindfulness meditation, and providing platforms for artistic expression where talents are showcased and acknowledged. In summary, AI not only enriches the spiritual life of deaf undergraduate students but also creates a favorable environment conducive to their mental health and personal development, effectively elevating their overall levels of positive emotions.

## Data Availability

The datasets presented in this study can be found in online repositories. The names of the repository/repositories and accession number(s) can be found in the article/supplementary material.

## Footnotes

^1^https://www.wjx.cn
